# Mimicking Hypoxia to Treat Anemia: HIF-Stabilizer BAY 85-3934 (Molidustat) Stimulates Erythropoietin Production without Hypertensive Effects

**DOI:** 10.1371/journal.pone.0111838

**Published:** 2014-11-13

**Authors:** Ingo Flamme, Felix Oehme, Peter Ellinghaus, Mario Jeske, Jörg Keldenich, Uwe Thuss

**Affiliations:** 1 Cardiology/Hematology, Acute Care Research, Global Drug Discovery, Bayer Pharma AG, Wuppertal, Germany; 2 Biotech Development, Global Biologics, Bayer Pharma AG, Wuppertal, Germany; 3 Clinical Science, Global Biomarkers, Bayer Pharma AG, Wuppertal, Germany; 4 Global Chemical Product Development, Bayer Pharma AG, Wuppertal, Germany; 5 Drug Metabolism and Pharmacokinetics, Global Early Development, Bayer Pharma AG, Wuppertal, Germany; Center for Molecular Biotechnology, Italy

## Abstract

Oxygen sensing by hypoxia-inducible factor prolyl hydroxylases (HIF-PHs) is the dominant regulatory mechanism of erythropoietin (EPO) expression. In chronic kidney disease (CKD), impaired EPO expression causes anemia, which can be treated by supplementation with recombinant human EPO (rhEPO). However, treatment can result in rhEPO levels greatly exceeding the normal physiological range for endogenous EPO, and there is evidence that this contributes to hypertension in patients with CKD. Mimicking hypoxia by inhibiting HIF-PHs, thereby stabilizing HIF, is a novel treatment concept for restoring endogenous EPO production. HIF stabilization by oral administration of the HIF-PH inhibitor BAY 85-3934 (molidustat) resulted in dose-dependent production of EPO in healthy Wistar rats and cynomolgus monkeys. In repeat oral dosing of BAY 85-3934, hemoglobin levels were increased compared with animals that received vehicle, while endogenous EPO remained within the normal physiological range. BAY 85-3934 therapy was also effective in the treatment of renal anemia in rats with impaired kidney function and, unlike treatment with rhEPO, resulted in normalization of hypertensive blood pressure in a rat model of CKD. Notably, unlike treatment with the antihypertensive enalapril, the blood pressure normalization was achieved without a compensatory activation of the renin–angiotensin system. Thus, BAY 85-3934 may provide an approach to the treatment of anemia in patients with CKD, without the increased risk of adverse cardiovascular effects seen for patients treated with rhEPO. Clinical studies are ongoing to investigate the effects of BAY 85-3934 therapy in patients with renal anemia.

## Introduction

The glycoprotein erythropoietin (EPO) is an indispensable growth factor for the production of red blood cells in the bone marrow. EPO is mainly secreted by the kidney but also, to a small degree in adults, by the liver. Anemia is a frequent complication of chronic kidney disease (CKD) because failing kidneys produce insufficient EPO to maintain normal red blood cell levels and hepatic EPO production cannot compensate [1]. Since its introduction into clinical use in 1989, recombinant human EPO (rhEPO) has become the standard therapy for anemia associated with renal failure [2]. However, treatment with rhEPO may be associated with an increased risk of cardiovascular events [3]. The chronic, intermittent treatment regimen can result in rhEPO levels that greatly exceed the normal physiological range for endogenous EPO. This could contribute to the increased blood pressure observed in patients with CKD treated with rhEPO because EPO has been found to directly induce endothelial dysfunction in resistance arteries in patients with CKD [4]–[6]. Therefore, it is highly desirable to develop alternative therapies to rhEPO that have equivalent efficacy in the treatment of anemia while avoiding excessive plasma EPO levels.

The expression of EPO in response to hypoxia is the accepted paradigm of oxygen-regulated gene expression. Systematic analysis of EPO gene regulatory elements led to the discovery of the hypoxia-inducible factors (HIFs), HIF-1 and HIF-2, which are constituents of the common oxygen-sensing pathway that enables higher organisms to cope with changes in oxygen supply [7], [8]. HIFs are the transcriptional activators of a plethora of hypoxia-inducible genes. The pattern of target gene response facilitates the homeostasis of oxygen supply by adjusting the levels of oxygen-carrying erythrocytes and regulating angiogenesis, thereby enabling metabolic adaptation to changing oxygen levels.

HIFs are heterodimers consisting of an α- and a β-subunit, which bind to distinct hypoxia-responsive elements in the regulatory sequences of hypoxia-inducible genes. Whereas HIF-β is constitutively expressed, the availability of HIF-α is under the control of a family of three enzymes, the HIF prolyl hydroxylases (also known as prolyl hydroxylase domain-containing protein 1–3, PHD1–3 or C. elegans EGL9 homolog 1–3, EGLN1–3) [9], [10]. HIF-PHs are oxygen-dependent and 2-oxoglutarate-consuming dioxygenases that, in the presence of oxygen, hydroxylate the HIF-α subunits at two distinct proline residues, thereby tagging them for polyubiquitination and proteasomal degradation [11], [12]. An E3 ubiquitin ligase protein complex consisting of the von Hippel–Lindau protein and elongin B and C (VBC complex) recognizes the hydroxylated HIF-α subunits and is required for the degradation of HIFs under normoxia [13]. Human genetic data suggest that renal EPO gene expression is under the non-redundant control of the PHD2–HIF-2α axis. A mutation in von Hippel–Lindau protein (Arg200Trp) that affects the interaction with hydroxylated HIFs, and loss- and gain-of-function mutations of the PHD2 and HIF-2α genes, respectively, have been identified as the underlying causes for rare forms of benign polycythemia at inappropriately high EPO levels. In contrast to patients with other forms of polycythemia, there is no tendency to develop arterial hypertension [14], [15]. The phenotype has been reproduced in transgenic mice and is in support of earlier observations that the increase in EPO transcription *in vivo* is by far the most sensitive response to hypoxia in the kidney [16]–[20].

Conversely, small increases in the availability of oxygen to EPO-producing cells (located in the peritubular interstitium) may be followed by critically reduced EPO transcription. This is the case in renal failure, when a reduced glomerular filtration rate and tubular reabsorption result in decreased oxygen utilization [21]. In concert with pro-inflammatory cytokines and insufficient clearance of uremic toxins, the reduced EPO production provides the basis for renal anemia [1], [22]. However, although patients with CKD and anemia present with low serum EPO levels, their kidneys are still able to produce EPO in response to a hypoxic stimulus [23]. This has also been shown in rats with gentamicin-induced renal anemia [24]. Compared with controls, higher oxygen tension in failing kidneys has been directly demonstrated in the rat remnant kidney (subtotal nephrectomy) model, which is commonly used as a model for CKD that comprises proteinuria, uremia, reduced EPO production with anemia, and increased blood pressure [25].

Data from epidemiological studies also support the hypothesis that hypoxia can overcome the EPO arrest in failing kidneys. Patients with end-stage renal disease (ESRD) living at altitude (above 6,000 feet) and undergoing hemodialysis required less rhEPO, but had higher hematocrit levels, than patients with ESRD living at sea level and undergoing hemodialysis [26]. Similarly, patients with ESRD showing poor treatment response at baseline who moved to a dialysis center at altitude showed increases in hematocrit and decreases in rhEPO requirements relative to a control group [27].

HIF-PH inhibitors provide a novel therapeutic approach to the treatment of anemia that is based on mimicking the hypoxia-driven expression of endogenous EPO in the kidney [28], [29]. Here, we present for the first time a comprehensive pharmacological characterization of a novel HIF-PH inhibitor (BAY 85-3934, molidustat), ranging from its activity *in vitro* to its effects in healthy rats and monkeys and in models of impaired kidney function. Our preclinical data confirm that, by pharmacological inhibition of HIF-PH, the failing kidney can be stimulated to produce sufficient EPO to correct for renal anemia, and that in contrast to treatment with rhEPO, adverse hypertensive effects are not only avoided, but CKD-associated hypertension may be ameliorated by this new therapeutic approach.

## Materials and Methods

### Compounds and reagents

BAY 85-3934, 2-[6-(morpholin-4-yl)pyrimidin-4-yl]-4-(1H-1,2,3-triazol-1-yl)-1,2-dihydro-3H-pyrazol-3-one, was synthesized as described previously [30]. For *in vitro* experiments, BAY 85-3934 was prepared as a stock solution of 10 mM in DMSO. For oral administration in rats, BAY 85-3934 was prepared as a solution in ethanol:Solutol HS 15:water (10∶20∶70), and for oral administration in cynomolgus monkeys as a solution in 0.5% tylose. The compound was administered in a volume of 2–5 ml/kg body weight and control animals received equal volumes of the vehicle. Before administration, the formulation was freshly prepared in water from a stock solution in ethanol/Solutol HS 15 stored at –20°C. Human recombinant EPO (Ortho Biotech) was administered via s.c. injection twice weekly at a dose of 100 IU/kg body weight, with saline (0.9% NaCl) as vehicle control.

In the chronic disease models of subtotal nephrectomy and inflammation, the sodium salt of BAY 85-3934 was used. For administration in drinking water, BAY 85-3934 sodium and enalapril were prepared as 100 mM sodium bicarbonate saline solutions (adjusted to pH 7) at final concentrations of 80 ppm and 30 ppm, respectively. Sham and vehicle control animals received saline only. Unless otherwise stated, chemicals and reagents were purchased from Sigma-Aldrich.

### Protein expression and purification

Recombinant human HIF-PHs were purified from Sf9 insect cell lysates. After infection with the recombinant virus stocks, generated via cloning of the respective cDNAs into pBacPAC transfer vectors (Clontech Laboratories), Sf9 cells were incubated for 48 h at 27°C with continuous shaking. Expression of the recombinant protein was confirmed by SDS-PAGE and western blot analysis of the expressed proteins. The lysate was centrifuged at 75,000 *g* at 4°C for 30 min and the supernatant containing the soluble HIF-PHs was incubated for 1 h at 2–8°C with CM Sepharose. After several wash steps, bound proteins were eluted from Sepharose using a linear NaCl gradient. Fractions containing HIF-PH activity were pooled, dialyzed against 100 mM Tris, 1.5 mM MgCl_2_, 2 mM DTT, 0.01% Tween-20, supplemented with 1% BSA and complete protease inhibitor cocktail without EDTA (Roche Diagnostics), and stored at –80°C. The VBC complex was expressed in *Escherichia coli* BL21(DE3) pLysS cells using the plasmid pST39-HisTrxNVHL-elongin B-elongin C. Protein expression and purification were performed as described previously [31]. DELFIA labeling reagent (PerkinElmer) was used to label purified VBC complex with europium according to the manufacturer’s instructions.

### Prolyl hydroxylase assay

The prolyl hydroxylase assay was performed as described previously [32] with minor modifications. Biotinylated HIF-1α 556–574 (biotinyl-DLDLEMLAPYIPMDDDFQL) was bound to white 96-well NeutrAvidin high binding capacity plates (Pierce Biotechnology), which were pre-blocked with Blocker Casein (Pierce Biotechnology) and subsequently blocked with 1 mM biotin. The immobilized peptide substrate was incubated with the appropriate amount of HIF-PH in buffer containing 20 mM Tris (pH 7.5), 5 mM KCl, 1.5 mM MgCl_2_, 20 µM 2-oxoglutarate, 10 µM FeSO_4_, 2 mM ascorbate, 4% protease inhibitors without EDTA (Roche Diagnostics) in a final volume of 100 µl, with or without test compound added at appropriate concentrations. The reaction time was 60 min. To stop the reaction, plates were washed three times with wash buffer.

Hydroxylated biotinyl-HIF-1α 556–574 was incubated with Eu-VBC in 100 µl binding buffer (50 mM Tris [pH 7.5], 120 mM NaCl) for 60 min at room temperature. After washing six times with DELFIA wash buffer (PerkinElmer) and adding 100 µl enhancer solution (PerkinElmer), the amount of bound VBC was determined by measuring time-resolved fluorescence with a Tecan infinite M200 plate reader. Measurements were taken in triplicate or more, and results were expressed as means ± SEM. IC_50_ values were determined after curve fitting using GraphPad Prism software (GraphPad Software, La Jolla, CA, USA) applying the four-parameter logistic equation to the data sets. When adjustment of the concentration of free Fe^2+^ was necessary, the reaction buffer was supplemented with appropriate amounts of ammonium iron(II) sulfate ((NH_4_)_2_Fe(SO_4_)_2_.6H_2_O, Mohr’s salt).

### Cell lines, cell culture media, and luciferase reporter assay

A549 and HeLa carcinoma cell lines (American Type Culture Collection) were cultured in DMEM/F-12 (Gibco), and Hep3B cells in RPMI medium, both supplemented with antibiotics, L-glutamine and 10% fetal calf serum. A549 cells stably transfected with the HIF-RE2-luc HIF reporter construct (constructed in pGL3, Promega GmbH) were seeded on 384-well plates (Greiner) at a density of 2500 cells/well in a volume of 25 µl complete cell culture medium, and re-incubated for 16–24 h before the test [33]. Test compounds were added at appropriate dilutions in a volume of 10 µl, and cells were re-incubated for 6 h before measurement. Luciferase activity was determined in a luminometer after addition of cell lysis/luciferase buffer. Cell line identities were verified by STR DNA typing (DSMZ GmbH).

### Western blot analysis

For western blot analysis, cell lysates were separated on 4–12% SDS polyacrylamide gradient gels (Invitrogen). Proteins were blotted onto polyvinylidene difluoride (PVDF) membranes (Amersham Biosciences). HIF-1α protein was detected using a HIF-1α specific monoclonal antibody (BD Transduction Laboratories) at a dilution of 1∶250. HIF-2α protein was detected using a HIF-2α specific polyclonal antibody (Novus Biologicals) at a dilution of 1∶1000. Anti-β-actin antibody served as a loading control. Binding of the antibodies was visualized by binding of a horseradish peroxidase-conjugated anti-mouse IgG antibody (Amersham Pharmacia Biotech), and subsequently enhanced using chemiluminescence (Chemiluminescent Peroxidase Substrate), according to the manufactureŕs instructions. Novex Sharp Pre-stained Protein Standard (Invitrogen) was used as molecular weight marker.

### Studies in animals

All procedures conformed to national legislation (dt. Tierschutzgesetz v. 18.05.2006) and EU directives (86/609) for the use of animals for scientific purposes and were approved by the institutional animal care office of Bayer AG and by the competent regional authority (LANUV Recklinghausen). All surgical procedures were performed under deep anesthesia (2% isoflurane) with immediate post-operative analgesia (ketamine/carprofen), and all efforts were made to minimize suffering. Rats were sacrificed by exposure to 10% isoflurane and consecutive cervical dislocation. Standard laboratory diet and tap water were available ad libitum. Rats were provided with wood sticks for chewing and gnawing, and monkeys were provided with appropriate toys such as balls and climbing bars. Animals were housed in temperature- and humidity-controlled cages with a 12 h light/dark cycle. In each experiment, the number of animals used was minimized. Animals were either randomly assigned to experimental groups or, if appropriate, were assigned based on hematocrit levels.

### Studies in rats

Male Wistar rats (240–340 g in body weight) were housed with five animals per cage for at least 1 week before experimentation. Blood samples from rats were collected under anesthesia (2% isoflurane in air) by puncturing the retro-orbital vein plexus with a glass capillary. In a repeat-dose, 26-day experiment, animals were administered vehicle or BAY 85-3934 at doses of 0.5 mg/kg, 1.25 mg/kg, 2.5 mg/kg, and 5 mg/kg. PCV was determined at baseline and at weekly intervals after centrifugation in a hematocrit capillary tube (Brand) for 10 min at full speed in a Haemofuge centrifuge (Heraeus). The number of reticulocytes in 5 µl blood was counted after staining with thiazol orange (Becton Dickinson) according to the manufacturer’s instructions by FACS analysis on a BD FACSCalibur system (Becton Dickinson). The efficacy of BAY 85-3934 (2.5 mg/kg, once-daily, oral) was also compared with that of rhEPO (25 IU/kg, 50 IU/kg, and 100 IU/kg, twice-weekly, s.c. injection). The time-course of induction of EPO mRNA expression and plasma EPO was determined at baseline and 0.5 h, 1 h, 2 h, 4 h, 6 h, and 8 h after oral administration of a single dose of BAY 85-3934 (5 mg/kg).

### Studies in cynomolgus monkeys

Male and female cynomolgus monkeys (2.8–5.6 kg in body weight) were used, which were housed two per cage. Blood samples from conscious cynomolgus monkeys were taken by puncturing a superficial vein. In a 5-day, repeat-dose study of plasma EPO response, BAY 85-3934 was administered at doses of 0.5 mg/kg and 1.5 mg/kg at 0 h, 24 h, 48 h, 72 h, and 96 h. Blood samples were taken at 7 h, 31 h, 55 h, 79 h, 103 h, and 168 h. Erythropoietic parameters were also evaluated after a 2-week treatment period with s.c. administration of rhEPO (100 IU/kg twice weekly at days 1, 4, 8, and 11) and BAY 85-3934 (1.5 mg/kg) once daily.

### Gentamicin-induced kidney failure model

Male Wistar rats were treated once daily with gentamicin (Gibco/Invitrogen) at a dose of 100 mg/kg body weight via i.p. injection on 14 consecutive days [34], [35]. Control animals received injections of an equal volume of 0.9% saline. After gentamicin treatment, PCV was determined and animals were distributed to the vehicle or treatment groups with respect to equal mean PCV. On day 15, BAY 85-3934 was given orally once daily at doses of 1 mg/kg, 2.5 mg/kg, 5.0 mg/kg, and 10.0 mg/kg, five times weekly.

### PG-PS-induced inflammatory anemia model

Female Lewis rats (Harlan Laboratories), with a body weight of 155–181 g were used. Body weight, ankle diameter, hematocrit, and blood cell count were determined at baseline and thereafter at regular intervals. PG-PS from *Streptococcus pyogenes* (Becton Dickinson) was dissolved in sterile saline and administered via i.p. injection at 15 mg/kg. Animals that did not show an inflammatory response were not studied further. Two weeks after injection, animals were distributed into treatment groups in equal proportions based on their hematocrit levels. On day 15, BAY 85-3934 was given orally once daily at doses of 2.5 mg/kg and 5.0 mg/kg. At the end of the study, animals were sacrificed and kidney and liver samples were processed for qRT-PCR analysis.

### Subtotal nephrectomy model

Subtotal nephrectomy was conducted in adult male Wistar rats. Body weight, blood pressure, hematocrit, and blood cell counts were determined at baseline and thereafter at weekly intervals. At baseline, rats were randomly distributed into two groups: those that underwent subtotal nephrectomy and those that underwent a sham procedure without reduction of renal mass. Surgery was performed in deeply anesthetized (2% isoflurane in air) animals. Kidneys were accessed via a dorsolateral incision of the body wall of about 2 cm in length. The right kidney was removed after ligature of the renal peduncle, and subsequently the upper and lower pole of the left kidney were removed, followed by careful hemostasis. Approximately one third of the initial kidney mass remained (removed tissue was weighed to check this was achieved). In the sham-treated animals, both kidneys were exposed before closure of the wound. Three weeks after surgery, animals were allocated to each group in equal proportions with respect to systolic blood pressure and hematocrit values. For 5 weeks, animals were treated twice weekly with rhEPO (100 IU/kg), or once daily with BAY 85–3936 sodium (2.5 mg/kg or 5.0 mg/kg) or vehicle. In experiments using enalapril or a combination of BAY 85-3934 sodium and enalapril, study drugs were administered with drinking water. BAY 85-3934 sodium and enalapril were administered in drinking water at concentrations of 80 ppm and 30 ppm, respectively. This was equivalent to approximately 2 mg/kg/day for enalapril and 5 mg/kg/day for BAY 85-3934. Systolic blood pressure and heart rate were determined using the tail-cuff method (a semi-automatic, non-invasive blood pressure monitor; TSE Systems), with three repeated measurements per animal.

### Blood analytics

EPO was measured in plasma samples taken from rodents using a commercial ELISA kit (R&D Systems), according to the manufacturer’s instructions. Relative plasma EPO levels were given in picograms per milliliter. In cynomolgus monkeys, EPO was measured using a combination anti-human EPO ELISAs (R&D Systems and Roche Diagnostics), with human EPO as a standard [36]. Values were given as units per liter. For determination of plasma prorenin from rats, a commercial ELISA (Oxford Biomedical Research) was used according to the manufacturer’s instructions. Hemoglobin in rodent blood samples was determined on a Cell-Dyn 3700 apparatus (Abbott Diagnostics). BAY 85-3934 plasma concentrations were determined after protein precipitation with acetonitrile/ammonium acetate using an internal standard, followed by separation using HPLC/mass spectrometry.

### RNA extraction and qRT-PCR

Total RNA was extracted from shock-frozen cell or tissue samples using the TRIzol method. Integrity of obtained RNA was checked on a Bioanalyzer (Agilent Technologies). For reverse transcription, 1 µg of total RNA was digested with RNase-free DNase I (Gibco) for 15 min at room temperature, and then reverse-transcribed using Promiscript (Promega GmbH) in a total reaction volume of 40 µl according to the standard protocol of the supplier. After inactivation of the enzyme by heating to 65°C for 15 min, the obtained cDNA was diluted to a final volume of 150 µl with double-distilled water, and 4 µl used per PCR reaction. qRT-PCR, including normalization of raw data to cytosolic β-actin, was carried out as described previously [37]. The resulting expression is given in arbitrary units. The sequences of the oligonucleotide primers and probes used are given in Table 1.

**Table 1:** Oligonucleotide primers and probes used for quantitative RT-PCR analysis of samples from rat tissues, and human cell lines (in italics).

Symbol	Gene name	Forward primer	Probe	Reverse primer
ADM	Adrenomedullin	CGCAGTTCCGAAAGAAGTGG	TAAGTGGGCGCTAAGTCGTGGGAAGAG	CCCGTAGGGTAGCTGCTGGA
		*CGCCAGAGCATGAACAACTTC*	*TGGCACACCAGATCTACCAGTTCAC*	*GCGACGTTGTCCTTGTCCTT*
ANGPTL-4	Angiopoietin-like 4	CTGGGTGCCACCAATGTTTC	CCCAATGGCCTTTCCCTGCCCT	CGTGGTCTTGGTCCCAGGTA
		*GGCCTCTCCGTACCCTTCTC*	*TCACGACCTCCGCAGGGACAA*	*AGAGGCTCTTGGCGCAGTT*
Beta-actin	Beta-actin	ACCTTCAACACCCCAGCCA	ACGTAGCCATCCAGGCTGTGTTGTCC	CAGTGGTACGACCAGAGGCA
		*TCCACCTTCCAGCAGATGTG*	*ATCAGCAAGCAGGAGTATGACGAGTCCG*	*CTAGAAGCATTTGCGGTGGAC*
CAIX	carbonic anhydrase IX	TCGTCTGGAGCTCACCTCATT	CCGCACTCTGCAACCCCTGGAAC	CGGGAGAGACTGGAGCTCAT
		*ACCTGGTGACTCTCGGCTACA*	*TGAACTTCCGAGCGACGCAGCCT*	*CCTCAATCACTCGCCCATTC*
eNOS	Endothelial nitricoxide synthase	CACACTGCTAGAGGTGCTGGAA	AATTTCCATCCGTGGCACTGCCTG	GGGTGAGGATCAGCGGG
EPO	Erythropoietin	CCGCTCCACTCCGAACAC	AGCGGATACTTTCTGCAAGCTCTTCCG	CCCGGAGGAAGTTGGAGTAGA
		*CCCCTCCAGATGCGGC*	*TCAGCTGCTCCACTCCGAACAATCAC*	*GAGTTTGCGGAAAGTGTCAGC*
GLUT-1	Glucose transporter-1	CAGCCTGTGTATGCCACCAT	TCGGGTATCGTCAACACGGCC	GACGAACAGCGACACCACAGT
		*AGCCTGTGTATGCCACCATTG*	*CCGGTATCGTCAACACGGCCT*	*GCTCGCTCCACCACAAACA*
HMOX-1	Heme oxygenase-1	TATCGTGCTCGCATGAACACT	TGGAGATGACCCCCGAGGTCAAGC	GGCGGTCTTAGCCTCTTCTGT
IGFBP-1	Insulin-likegrowth factorbinding protein-1	CTGCCAAACTGCAACAAGAA	TCACAGCAAACAGTGCGAGACATCTC	AGAGCCCAGCTTCTCCATC
IGFBP-3	Insulin-likegrowth factorbinding protein-3	TCCCAAACTGTGACAAGAAGG	AGAAACAGTGTCGCCCTTCCAAAGG	GTCCACGCACCAGCAGAA
		*CCACCCCCTCCATTCAAAG*	*TAATCATCATCAAGAAAGGGCATGCT*	*CTTTGTAGCGCTGGCTGTCTT*
LDH-A	Lactate dehydrogenase-A	ATTCGGCTCGGTTCCGTTA	CTGATGGGAGAAAGGCTGGGAGTTCA	CCACCCGTGACAGCTCAGT
PDK-1	Pyruvatedehydrogenaselipoamide kinase-1	CACCATGCAGACAAAGGCG	TCCCCCGATTCAAGTTCACGTCACA	AGTCAAATCCTCCTCCCCCA
PHD1	Prolyl hydroxylasedomain-containingprotein-1	TCTTTGACCGGTTGCTCATTT	TGGTCTGACCGACGGAATCCAC	GTGGCATAGGCTGGCTTCA
PHD2	Prolyl hydroxylasedomain-containingprotein-2	AAGCCCAGTTTGCTGACATTG	CCCAAGTTTGATAGATTGCTGTTTT	GAGGGTTACGCCGGTCAGA
PHD3	Prolyl hydroxylasedomain-containingprotein-3	CCCTCCTATGCCACCAGGTA	CATGACTGTCTGGTACTTCGATGCT	TCTTTTTGGCTTCTGCCCTTT
VEGF-A	Vascular endothelialgrowth factor-A	CGCAAGAAATCCCGGTTTAA	CTGGAGCGTTCACTGTGAGCCT	CAAATGCTTTCTCCGCTCTGA
		*AGCGGAGAAAGCATTTGTTTG*	*CAAGATCCGCAGACGTGTAAATGTTCCTG*	*CTTGCAACGCGAGTCTGTGT*

### Statistical analysis

Data are presented as the mean ± SEM. In dose–response experiments, statistical significance was evaluated by sequential application of a two-tailed t-test comparing compound-treated groups with corresponding controls. For inter-group comparisons, one-way ANOVA followed by Dunnett’s or Bonferroni’s multiple comparison was applied. A P value of <0.05 was considered statistically significant.

## Results

### Characterization of BAY 85-3934 *in* *vitro*

BAY 85-3934 resulted from the optimization of a compound identified by high-throughput screening. To measure the activity of HIF-PH inhibitors quantitatively, a microplate assay was developed that employed the interaction of the VBC complex with the peptide HIF-1α 556–574 after being hydroxylated at proline 564 by recombinant HIF-PH. Activity of the recombinant enzyme was dependent on the concentrations of several cofactors, 2-oxoglutarate, Fe^2+^, and ascorbate, in the reaction mixture. These concentrations were optimized to give a robust and highly reproducible assay [32].

**Figure 1:**
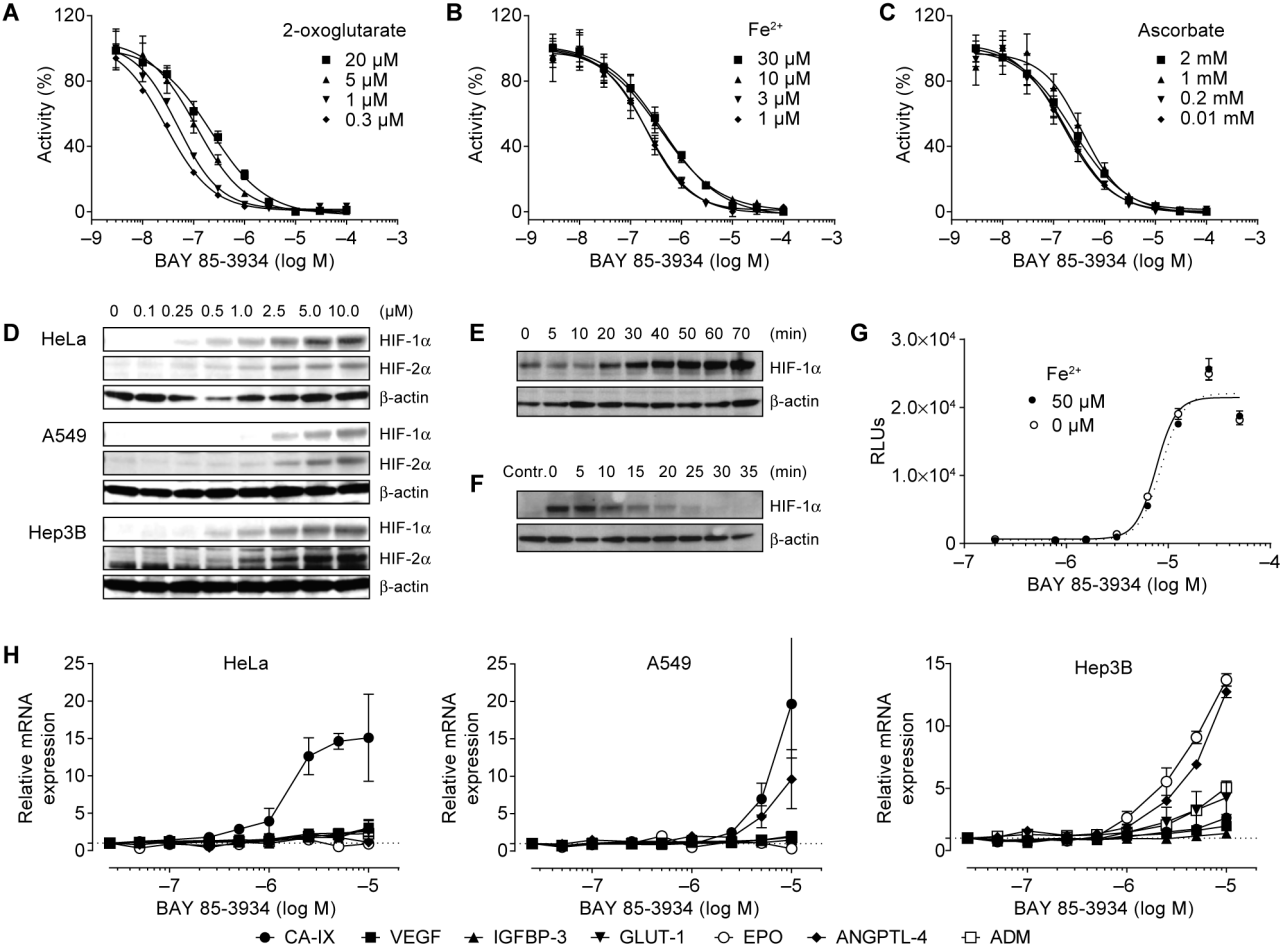
Characterization of the *in vitro* activity of BAY 85-3934. (A)–(C) Concentration–response curves of hypoxia-inducible factor prolyl hydroxylase (PHD2) activity for BAY 85–-934 after the addition of increasing concentrations of 2-oxoglutarate, Fe^2+^, and ascorbate. Data, presented as means ± SEM of 4 replicates, were normalized to basal activity without BAY 85-3934 (100%) and residual activity (0%). (D) Detection of HIF-1α and HIF-2α in HeLa, A549, and Hep3B cells by western blot analysis. (E) Time-course of induction of HIF-1α in HeLa cells after addition of serum-free medium containing BAY 85-3934 (5 µM). β-actin levels were measured as a loading control. (F) Time-course of disappearance of HIF-1α in A549 cells after induction with BAY 85-3934 (20 µM). Culture medium was withdrawn and replaced with medium containing cycloheximide (100 µM). β-actin levels were measured as a loading control (D–F show representative data of 3 independent experiments). (G) Concentration–response curve for luciferase activity of A549 HIF-RE2 reporter cells (relative luciferase units [RLUs]) after addition of BAY 85-3934 in the presence or absence of additional Fe^2+^. Data are presented as means ± SEM of 4 replicates. (H) Relative mRNA expression levels (means ± SD of 2 replicates) of a panel of HIF target genes (shown as fold-increase from baseline levels) after exposure to BAY 85-3934 in HeLa, A549, and Hep3B cells. For definition of gene symbols, see Table 1.

Under standard assay conditions, in the presence of 20 µM 2-oxoglutarate, 10 µM Fe^2+^, and 2 mM ascorbate, the mean IC_50_ values of BAY 85-3934 for PHD1, PHD2, and PHD3 were 480 nM, 280 nM, and 450 nM, respectively. The IC_50_ values were found to be dependent on the concentration of 2-oxoglutarate in the reaction buffer. By lowering the 2-oxoglutarate concentration from 20 µM to 0.3 µM, the potency of the test compound increased up to 10-fold (Fig. 1A). Variation of the concentrations of Fe^2+^ and ascorbate in the reaction buffer by factors of 30 and 200, respectively, did not alter the potency of the inhibitor by more than 2-fold (Fig. 1B, C). The IC_50_ values were always well below the concentration of Fe^2+^.

It was further explored whether inhibition of HIF-PHs would result in the stabilization of HIFs. Exposure of three different human cell lines (HeLa, A549, and Hep3B) to concentrations of BAY 85-3934 up to 10 µM for 2 h led to dose-dependent increases in both HIF-1α and HIF-2α. Within the individual cell lines, there was no difference between the HIF isoforms with regard to the threshold concentration of BAY 85-3934 that was necessary for the induction of detectable amounts of HIF. However, HeLa cells, which had an induction threshold of 0.25 µM of BAY 85-3934, appeared to be more sensitive than Hep3B and A549 cells, which had induction thresholds of 0.5 µM and 2.5 µM, respectively (Fig. 1D). Exposure of HeLa cells to 5 µM BAY 85-3934 for 20 min was sufficient to induce detectable concentrations of HIF-1α (Fig. 1E). After exposure of A549 cells to 20 µM BAY 85-3934 for 120 min, with subsequent withdrawal of the culture medium and replacement with medium containing 100 µM cycloheximide (an inhibitor of protein synthesis), induction of HIF-1α was no longer detectable after 30 min (Fig. 1F). Therefore, it can be ruled out that disappearance of HIF-1α was the result of de novo synthesis of HIF-PH.

In a cellular reporter assay, BAY 85-3934 induced the expression of the firefly luciferase reporter gene under the control of a hypoxia responsive element promoter at a mean (± SD) EC_50_ of 8.4± 0.7 µM (*n* = 4). As in the enzymatic assay, and in contrast to experiments with iron-chelating agents (data not shown), luciferase activity was not altered by a high concentration of Fe^2+^ (50 µM) in the culture medium (Fig. 1G). The high IC_50_ value observed corresponds well to the low sensitivity of A549 cells shown by western blot analysis.

Finally, we investigated whether BAY 85-3934 induced the transcription of hypoxia-sensitive genes in HeLa, A549, and Hep3B cells. The mRNA expression levels of a panel of HIF target genes were analyzed by quantitative RT-PCR (qRT-PCR) in the three cell lines 4 h after exposure to concentrations of BAY 85-3934 up to 10 µM. mRNA levels were found to be induced by BAY 85-3934 in a dose-dependent manner. The basal level of expression and the factor of induction varied considerably between cell lines. The highest induction factors (up to 20-fold) were observed for carbonic anhydrase IX (CA-IX) in HeLa cells, CA-IX and angiopoietin-like 4 (ANGPTL-4) in A549 cells, and EPO and ANGPTL-4 in Hep3B cells (Fig. 1H). The other genes examined were induced between 2- and 5-fold. The mRNA expression level of ANGPTL-4 was not increased by BAY 85-3934 in HeLa cells. EPO mRNA was not detectable at baseline and not induced in HeLa or A549 cells. The threshold concentration for induction of CA-IX expression was 500 nM in HeLa cells, and greater than 1 µM for all other genes that showed a response.

The selectivity of BAY 85-3934 was examined via a broad panel of radio-ligand binding assays (*n* = 67), and by examining the activity of BAY 85-3934 against related enzymes (matrix metalloproteinases and other peptidases, *n* = 8). At a concentration of 10 µM, no significant (> 50%) activity in any of these assays was detected (data not shown). BAY 85-3934 was found to have pharmacokinetic properties suitable for oral dosing studies in rats and monkeys.

### Induction of EPO and erythropoiesis in male Wistar rats

**Figure 2:**
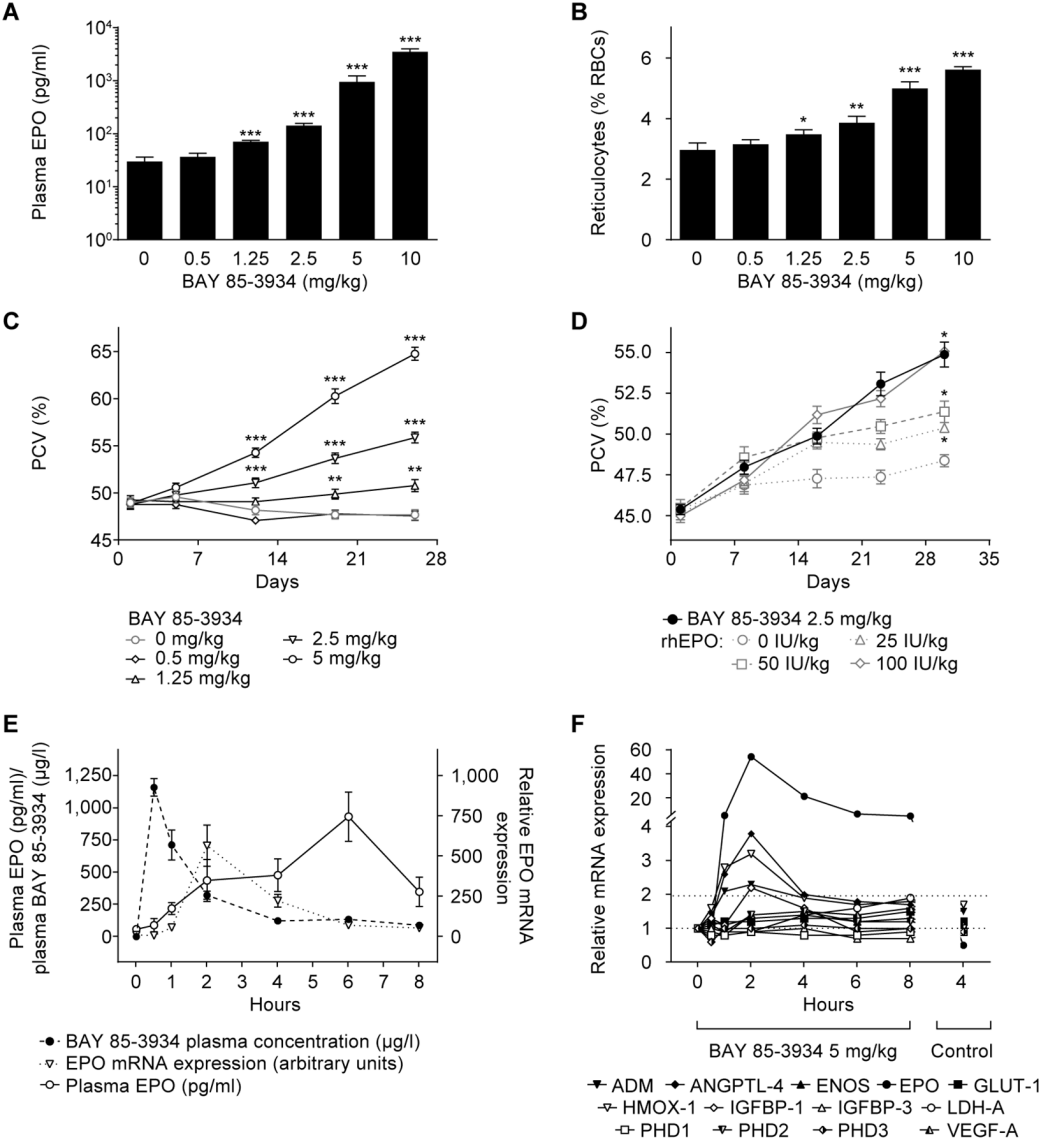
Characterization of the *in vivo* activity of BAY 85-3934 (in male Wistar rats). Data are presented as means ± SEM. (A) Increase in plasma erythropoietin (EPO) at 4 h and (B) reticulocytes (as a proportion of red blood cells [RBCs]) at 72 h following single oral dosing of BAY 85-3934. Data were pooled from two sequential experiments (*n* = 2×5 animals per group). *p<0.05, **p<0.01, and ***p<0.001; unpaired t-test, sequentially pairwise-applied to dose groups and corresponding vehicle group. (C) Change in packed cell volume (PCV) during once-daily dosing with BAY 85-3934 (*n* = 12 animals per group). **p<0.01 and ***p<0.001; two-way ANOVA with Dunnett’s multiple comparison test versus vehicle group. (D) Induction of erythropoiesis after subcutaneous administration of recombinant human EPO (rhEPO) twice weekly or BAY 85-3934 (2.5 mg/kg) once daily (*n* = 10 animals per group). *p<0.001 compared with control (t-test) at day 30. (E) BAY 85-3934 plasma levels, kidney EPO relative mRNA expression, and plasma EPO levels after oral administration of BAY 85-3934 (5 mg/kg) (*n* = 5 animals per group). (F) Relative mRNA expression levels of HIF target genes in rat kidney after administration of BAY 85-3934 (5 mg/kg). Baseline expression was set at 1 (*n* = 5 animals per group; error bars not show for clarity of presentation). For definition of gene symbols, see Table 1.

Experiments were conducted in male Wistar rats to assess the threshold doses for EPO induction and the erythropoietic activity of BAY 85-3934. Following a single oral administration of BAY 85-3934, EPO was statistically significantly induced at doses of 1.25 mg/kg and above (Fig. 2A). EPO induction was maximal at doses of 500 mg/kg and above, and these plasma concentrations were maintained for over 48 h following administration (data not shown). Induction of EPO after single-dose administration was followed by a dose-dependent increase in the proportion of reticulocytes at doses of 1.25 mg/kg and above (Fig. 2B).

In a 26-day, repeat-dose experiment, treatment with BAY 85-3934 resulted in a dose-dependent increase in mean packed cell volume (PCV; i.e. hematocrit) (Fig. 2C). A significant elevation of mean PCV from that in the vehicle control was observed from a dose of 1.25 mg/kg, which resulted in a gain of approximately 3% in mean PCV by day 26. At the highest dose (5 mg/kg), a mean PCV gain of 17% from baseline was observed. BAY 85-3934 was well tolerated, and body weight increased normally in animals in all dose groups.

The efficacy of oral treatment with BAY 85-3934 was compared with that of standard rhEPO treatment in male Wistar rats. All treatments resulted in a significant increase in hematocrit compared with the control group (*p*<0.001) (Fig. 2D). Over time, the mean gains in hematocrit and hemoglobin were very similar for the BAY 85-3934 treated group and for rats that received the highest dose of EPO. Therefore, in this model, the once-daily dose of BAY 85-3934 (2.5 mg/kg) can be considered equivalent to a twice-weekly dose of rhEPO (100 IU/kg).

The time-course of BAY 85-3934 plasma levels, induction of EPO mRNA expression, and plasma EPO was examined. In male Wistar rats, BAY 85-3934 (5 mg/kg) was rapidly absorbed, with a plasma maximum concentration (C_max_) of 30 min (Fig. 2E). The half-life was 3.4 h, and oral bioavailability was approximately 38%. The peak level of EPO mRNA expression was reached 2 h after administration (Fig. 2E). EPO plasma concentration reached a maximum at 6 h, at which point almost 90% of BAY 85-3934 had been eliminated from the plasma, and expression of EPO mRNA was approaching baseline levels (Fig. 2E).

The time-course of gene expression was further examined in the kidney for EPO and a panel of other HIF target genes following a single-dose administration of BAY 85-3934 (5 mg/kg). EPO mRNA expression was induced to about 50-fold over baseline, with a peak 2 h after administration (Fig. 2F). Of the other genes investigated, only heme oxygenase-1 (HMOX-1), insulin-like growth factor binding protein-1, adrenomedullin, and ANGPTL-4 showed significant induction of mRNA expression of more than 2-fold over baseline, with induction factors of 3.2-, 2.2-, 2.3-, and 3.8-fold, respectively (p<0.01). Peak levels were seen 2 h after administration. Induction of mRNA expression was moderate (less than 2-fold) or absent for the other genes examined.

### Repeat-dose studies in cynomolgus monkeys

**Figure 3:**
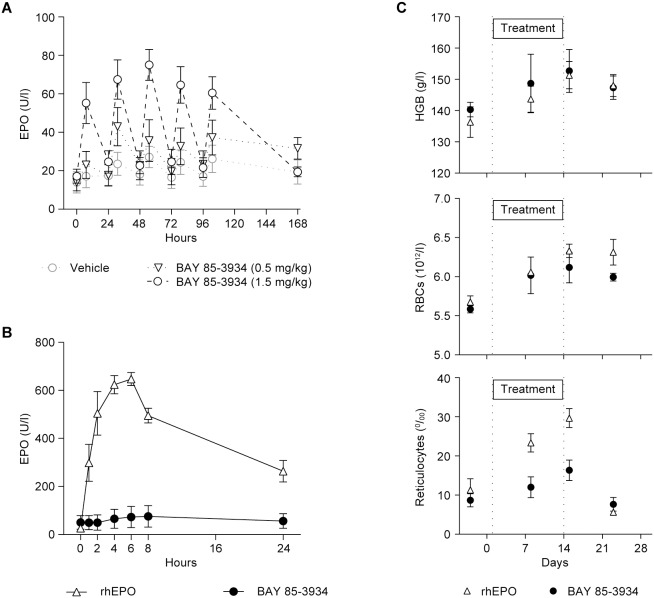
Effects of BAY 85-3934 or recombinant human erythropoietin (rhEPO) on erythropoietic parameters in cynomolgus monkeys. Data are presented as means ± SEM. (A) Plasma erythropoietin (EPO) concentrations after repeat oral administration of BAY 85-3934 (*n* = 6 animals per group). (B) Plasma EPO concentrations after a single s.c. administration of rhEPO (100 IU/kg) or a single oral dose of BAY 85-3934 (1.5 mg/kg) (*n* = 3 animals per group). (C) Erythropoietic parameters (hemoglobin [HGB], red blood cells [RBCs], and reticulocytes) after s.c. administration of rhEPO twice weekly (100 IU/kg) for 2 weeks or BAY 85-3934 (1.5 mg/kg) once daily for 2 weeks (*n* = 3 animals per group).

A multiple-dose study in cynomolgus monkeys was conducted to evaluate whether repeat administration of BAY 85-3934 would result in EPO accumulation and/or adaptation of the EPO response. EPO was significantly induced 7 h after administration of BAY 85-3934 (1.5 mg/kg) in all animals, and showed a clear increase after the 0.5 mg/kg dose in females (Fig. 3A). In all groups, EPO concentrations had returned to baseline concentrations 24 h after administration of the study drug. Notably, there was no adaptation of the EPO response after repeated dosing in any of the treated animals. Platelet and white blood cell counts were unchanged. With respect to EPO induction, the 0.5 mg/kg dose was determined as the minimal effective dose in this study, although statistical significance versus control animals was not reached in this small and heterogeneous animal cohort.

In a further study, the efficacy of oral treatment with BAY 85-3934 was evaluated in comparison with rhEPO. Animals were administered a single dose of rhEPO (100 IU/kg) by s.c. injection or BAY 85-3934 (1.5 mg/kg, oral). After administration of rhEPO, plasma concentrations of rhEPO were more than 8-fold higher than the endogenous EPO levels induced by treatment with BAY 85-3934 (Fig. 3B). The area under the curve (AUC) of rhEPO was more than 6-fold higher than the AUC of endogenous EPO. However, in a 14-day repeat-dose study, both treatments resulted in mean gains of hemoglobin and red blood cells that were almost completely congruent over time (Fig. 3C). Mean reticulocyte counts were 2-fold higher after treatment with rhEPO than after treatment with BAY 85-3934, but returned to baseline values 8 days after treatment cessation.

### Erythropoietic efficacy of BAY 85-3934 in rats with gentamicin-induced renal anemia

**Figure 4:**
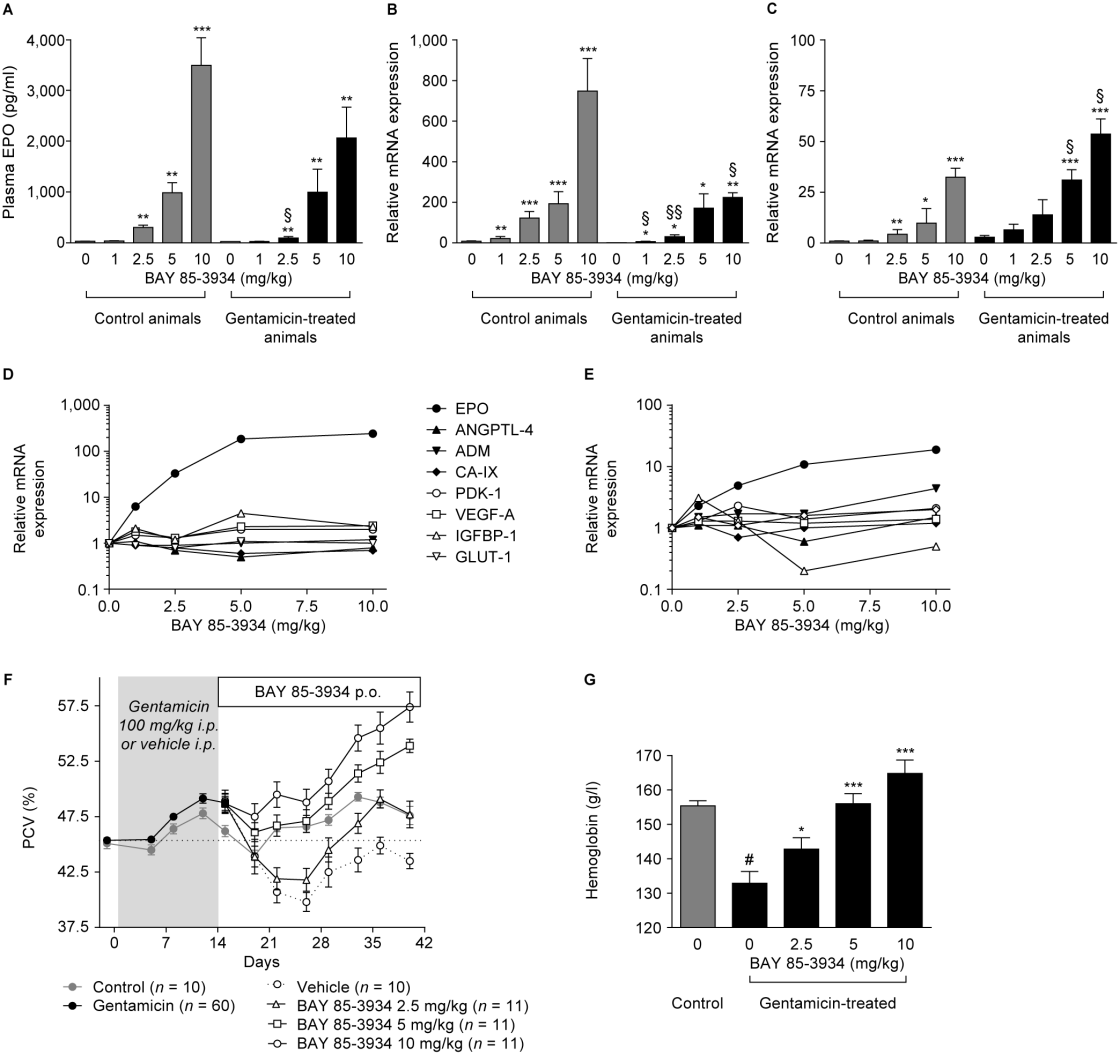
Effects of BAY 85-3934 administration in male Wistar rats treated with gentamicin to induce renal anemia. Data are presented as means ± SEM. (A) Plasma EPO levels, and (B) kidney and (C) liver relative expression of erythropoietin (EPO) mRNA 4 h after oral administration of BAY 85-3934. Before administration, rats had been treated with vehicle or gentamicin. (*n* = 5 animals per group). *p<0.05, **p<0.01, and ***p<0.001 compared with vehicle group,^ §^ *p*<0.05 and ^§§^ *p*<0.001 compared with control group, t-test. (D) Kidney and (E) liver mRNA expression levels of hypoxia-inducible factor target genes relative to mean of vehicle treated animals after oral administration of BAY 85-3934 (*n* = 4 to 5 animals per group, error bars not shown for clarity of presentation). For definition of gene symbols, see Table 1. (F) Time-course of changes in packed cell volume (PCV) following treatment with BAY 85-3934 or vehicle (once daily, five times per week, number of animals as indicated). (G) Hemoglobin levels 7 days after start of treatment with BAY 85-3934 or vehicle at day 22 of experiment shown in (F). *p<0.05, **p<0.01, and ***p<0.001 compared with vehicle group; ^#^ *p*<0.05 compared with sham group; t-test.

The effect of BAY 85-3934 on the induction of endogenous EPO production was evaluated in an animal model of impaired kidney function, the gentamicin-induced kidney failure model. A mean creatinine clearance of 30% of that of untreated animals and high expression levels of several markers of acute and chronic kidney injury were indicative of substantial renal impairment in these animals. Treatment with BAY 85-3934 significantly and dose-dependently induced plasma EPO levels (Fig. 4A). Accordingly, EPO mRNA expression increased dose-dependently in kidney samples taken from these animals (Fig. 4B). The mean absolute levels of induction were lower in the gentamicin-treated animals than in the controls.

Significant induction of EPO mRNA expression was detected in liver samples, in the control group at BAY 85-3934 doses of 2.5 mg/kg and above, and in gentamicin-treated animals at BAY 85-3934 doses of 5 mg/kg and above (Fig. 4C). As expected, the expression of EPO mRNA was considerably lower in liver samples than in kidney samples. Unlike the expression in the kidney, hepatic mRNA concentrations of EPO were slightly, but significantly, higher in gentamicin-treated animals than in hepatic samples from healthy (control) animals. In rats with renal impairment, it cannot be ruled out that the liver contributes significantly to plasma EPO concentrations after treatment with BAY 85-3934. However, in the absence of BAY 85-3934 treatment, the liver does not compensate for reduced renal EPO production.

In this study, the expression of other HIF target genes may also have been affected, although the magnitude of response was considerably lower than that seen for EPO mRNA expression in kidney and liver samples (Fig. 4D, 4E). Furthermore, unlike EPO mRNA expression, no dose-response was observed after administration of BAY 85-3934.

Treatment with BAY 85-3934 prevented the development of gentamicin-induced renal anemia. At the end of the study, rats treated with gentamicin and vehicle showed a lower mean PCV than control animals (Fig. 4F). However, treatment with BAY 85-3934 (5 mg/kg and 10 mg/kg) 5 days per week prevented the decline in mean PCV, with final values significantly higher than in control animals (*p*<0.001). Rats administered BAY 85-3934 (2.5 mg/kg) showed mean PCV levels similar to those in control animals, with mean PCV returning to baseline values within 2 weeks after onset of treatment with study drug. A significant dose-dependent increase in hemoglobin values was seen in animals treated with BAY 85-3934, as measured 7 days after the start of treatment (Fig. 4G). All doses led to an increase in reticulocyte counts, which were significantly higher than the increase associated with spontaneous recovery (data not shown).

### Erythropoietic efficacy of BAY 85-3934 in rodents with inflammatory anemia induced by peptidoglycan-polysaccharide (PG-PS)

Renal anemia, particularly in patients with ESRD on dialysis, is often associated with systemic inflammation and EPO resistance. The potential for BAY 85-3934 to reverse anemia was evaluated in a rodent model of protracted inflammatory anemia associated with chronic polyarthritis, induced by challenge with PG-PS [38]. The severity of the inflammatory response after PG-PS challenge was underscored by the finding that there was elevated expression of a panel of cytokines and inflammatory marker genes, several of which are known to disturb iron utilization (interleukin-1, interleukin-6, tumor necrosis factor-α, and hepcidin) and to impair proliferation and differentiation of erythroid precursors (interleukin-1 and tumor necrosis factor-α) [39].

**Figure 5:**
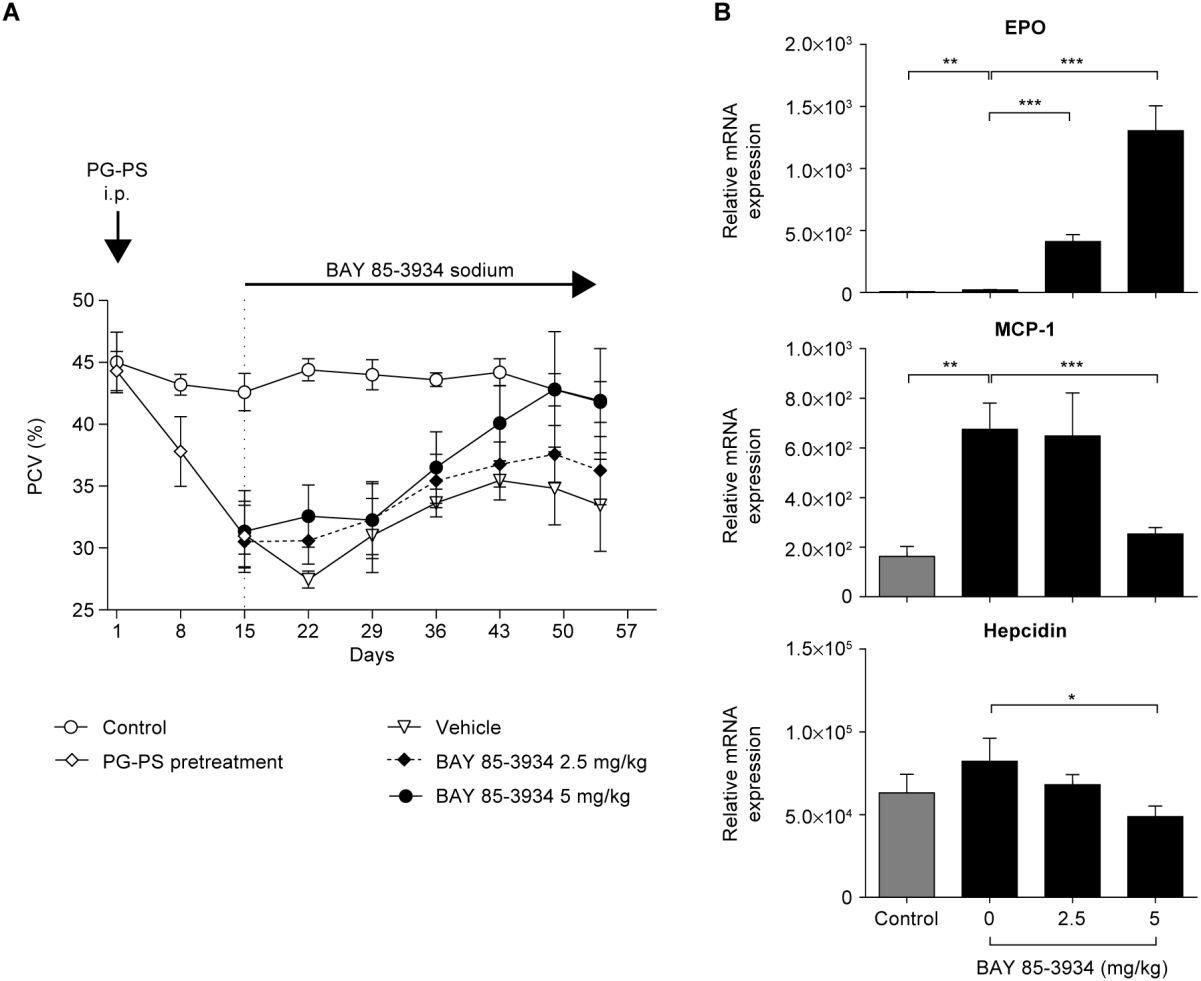
Effect of BAY 85-3934 administration on peptidoglycan-polysaccharide (PG-PS)-induced inflammatory anemia in female Lewis rats. Data are presented as means ± SEM. (A) Packed cell volume (PCV) in PG-PS-treated animals administered BAY 85-3934 or vehicle (*n* = 11–12 animals per group), compared with control animals treated with vehicle alone (*n* = 5 animals). (B) Relative expression of erythropoietin (EPO) and monocyte chemotactic protein-1 (MCP-1) mRNA in kidney, and hepcidin mRNA in liver at the end of the study. *p<0.05, **p<0.01, and ***p<0.001; t-test.

Daily treatment of rats with BAY 85-3934 (5 mg/kg) reversed mean PCV decline, and values returned to normal levels within 5 weeks of PG-PS challenge (Fig. 5A). The low dose of BAY 85-3934, 2.5 mg/kg, halted further decline of mean PCV levels, and by the end of the study, there was a trend for mean PCV values to be higher than in PG-PS-pretreated animals that received vehicle only. A small but significant increase in kidney EPO mRNA expression, interpreted as anemia-reactive, was not sufficient to counteract the inflammation-induced anemia in PG-PS-challenged animals. However, in response to treatment with BAY 85-3934, a significantly larger increase in EPO mRNA expression in the kidneys was observed. Among the inflammatory markers, the expression levels of monocyte chemotactic protein-1 and hepcidin were significantly reduced in animals in the 5 mg/kg dose group (Fig. 5B). Although treatment did not influence the course of polyarthritis, as monitored by hind limb ankle diameter and development of body weight gain, the initial increase in white blood cell count was attenuated (data not shown).

### Efficacy of BAY 85-3934 in a disease model of subtotal nephrectomy

As well as anemia and inflammation, CKD is often accompanied by hypertension, upon which treatment with rhEPO may have an unfavorable impact. The effects of BAY 85-3934 were compared with rhEPO in the remnant kidney (subtotal nephrectomy) model in rats. This commonly used model of CKD displays the triad of renal impairment, anemia, and hypertension.

**Figure 6:**
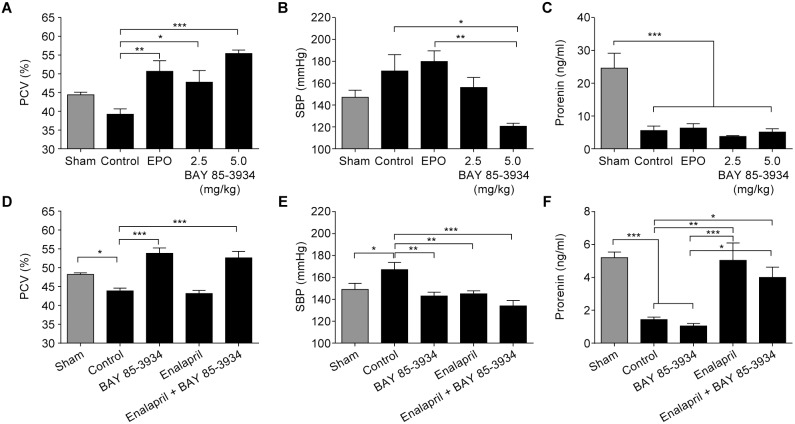
Effect of BAY 85-3934, erythropoietin (EPO), and enalapril in the rat subtotal nephrectomy model. Data are presented as means ± SEM. (A) Packed cell volume (PCV), (B) systolic blood pressure (SBP), and (C) prorenin following oral administration of BAY 85-3934 (2.5 mg/kg or 5 mg/kg once daily) or rhEPO (100 IU/kg s.c. twice weekly) for 5 weeks, compared with control and sham-operated animals (*n* = 4–6 animals per group). Efficacy of BAY 85-3934 sodium (80 ppm), enalapril (30 ppm), and a combination of both, administered in drinking water for 5 weeks, on (D) PCV, (E) SBP (at 4 weeks), and (F) prorenin (*n* = 9–10 animals per group). *p<0.05, **p<0.01, ***p<0.001; one-way ANOVA followed by Dunnett’s multiple comparison test to corresponding sham or control group for (A), (C), (D), and (E), and Bonferroni’s multiple comparison test to corresponding sham or control group for (B) and (F).

Subtotal nephrectomy resulted in a mild reduction in mean hematocrit, and an increase in mean systolic blood pressure as determined by tail cuff measurement (Fig. 6A, B). Treatment with rhEPO or BAY 85-3934 (2.5 mg/kg and 5 mg/kg) significantly increased mean PCV (Fig. 6A). In fact, 2 weeks after onset of treatment, animals in the 5 mg/kg group were switched to treatment with 2.5 mg/kg because their mean PCV had exceeded 50%.

After treatment with rhEPO, there was no difference in systolic blood pressure compared with untreated controls (Fig. 6B). In contrast, treatment with BAY 85-3934 led to a sustained reduction in mean systolic blood pressure, with almost normalized values in the 2.5 mg/kg group. Furthermore, systolic blood pressure was significantly lower in animals treated with BAY 85-3934 5 mg/kg than in control and rhEPO-treated animals. This was also mirrored by complete prevention of cardiac hypertrophy, which manifested in control animals and rhEPO-treated animals, but was absent in animals treated with BAY 85-3934 (data not shown). The mean plasma levels of prorenin were about 4-fold reduced in nephrectomized rats compared with sham-operated animals, which was consistent with the increased blood pressure observed (Fig. 6C). Unexpectedly, and unlike the case for other hypertensive treatments, the decrease in blood pressure after treatment with BAY 85-3934 was not followed by the return of prorenin to normal levels.

The effect of treatment with BAY 85-3934 was also compared with that of a standard antihypertensive treatment, the angiotensin-converting enzyme inhibitor enalapril. As expected, treatment with enalapril (administered at 30 ppm via drinking water) resulted in a significant reduction in mean systolic blood pressure compared with control, while mean PCV remained unchanged (Fig. 6D, E). Treatment with BAY 85-3934 sodium (administered at 80 ppm via drinking water) significantly reduced mean systolic blood pressure compared with controls, while increasing mean PCV. In normotensive rats, treatment with BAY 85-3934 at the same dose for 3 days did not affect blood pressure (data not shown). A combination of both treatments resulted in a slight reduction of mean systolic blood pressure; however, the difference was not significant compared with treatment with either study drug alone. In contrast to treatment with BAY 85-3934, the decrease in blood pressure with enalapril was accompanied by reversion of plasma prorenin levels to normal values (Fig. 6F). Compared with animals treated with enalapril alone, a small decrease in plasma prorenin levels was seen for animals treated with a combination of the two drugs.

In all of the experiments reported, no significant adverse events related to treatment with BAY 85-3934 were observed.

## Discussion

HIF-PH inhibitors are being evaluated in clinical studies as novel therapeutics for the treatment of anemia (e.g. FG-4592, AKB-6548, GSK1278863) [40]. They function as stabilizers of HIF, thereby mimicking the hypoxia-driven expression of endogenous EPO in the kidney [28]. To date, publications on pharmacological data for compounds under clinical development are still scant [29]. Here we present for the first time, a comprehensive pharmacological profile of a novel HIF-PH inhibitor under clinical development [41], [42], from *in vitro* characterization to animal models of kidney disease. It was demonstrated that BAY 85-3934 was a reversible, 2-oxoglutarate-competitive, pan-HIF-PH inhibitor with high selectivity against related enzymes (matrix metalloproteinases and other peptidases) and a broad panel of radio-ligand binding assays. There is, however, a lack of validated assays for other 2-oxoglutarate dependent dioxygenases. A fourth, putative HIF-PH (PH-4, P4H–TM) has been identified, located in the membrane of the endoplasmic reticulum that we and others have previously described [33], [43]. This enzyme is distantly related to PHDs, and evidence for its involvement in the hypoxia-dependent regulation of erythropoiesis in man is still elusive because, unlike for PHD2, no data from human genetics exists to help elucidate its role.

BAY 85-3934 consistently induced EPO and erythropoiesis after oral administration in rodent and non-rodent species. BAY 85-3934 was effective in animal models of renal and inflammatory anemia and, unlike rhEPO therapy, reduced blood pressure in a CKD model. Notably, the levels of endogenous EPO that were induced during treatment were close to the normal physiological range of EPO. This is in contrast to the standard therapy for renal anemia, whereby rhEPO levels exceed those normally seen for endogenous EPO.

BAY 85-3934 behaves as a hypoxia mimetic in the presence of oxygen both *in vitro* and *in vivo*, and HIF-1α and HIF-2α were dose-dependently and concordantly induced in human cell lines. With an induction threshold below 1 µM, BAY 85-3934 appears to be a potent cellular HIF stabilizer. These data compare favorably with those reported for other HIF-PH inhibitors [44]–[46]. The cellular effects are most likely to be due to direct modulation of HIF-PH, because BAY 85-3934 induced the stabilization of HIF-1α within 20 min after application. After withdrawal of the compound, stabilization of HIF-1α was completely reversed within 40 min. The kinetics of induction and disappearance of HIF-1α closely resemble those of HIF-1α in tonometer experiments after de-oxygenation and re-oxygenation [47]. Functionality of HIF stabilization was demonstrated in a hypoxia-sensitive luciferase reporter cell line, and by analysis of HIF target gene expression in human cell lines. Expression patterns were cell-line-specific, with a measurable response of EPO gene expression only in Hep3B cells. However, the drug concentrations needed to induce the transcriptional response *in vitro* were well above those that were effective for the induction of EPO *in vivo*. This could be the result of differences in cellular uptake mechanisms or oxygen levels, or could mirror the high sensitivity of renal EPO gene expression to hypoxia [18], [48].

In preclinical models and in a proof-of-concept study [41], oral administration of BAY 85-3934 was followed by a dose-dependent increase in EPO expression and subsequent erythropoiesis. EPO induction showed clear hysteresis with respect to plasma levels of the compound, and was transient. Accumulation of EPO or adaptation of the EPO response was not observed when administered once daily over 5 consecutive days to cynomolgus monkeys. This is in contrast to the rapid acclimatization of the EPO response that is observed at high altitude, which manifests as a decline in plasma EPO concentrations after 2 days [49].

Chronic, once-daily administration of BAY 85-3934 in cynomolgus monkeys led to increases in red blood cell production comparable to those achieved by chronic intermittent treatment with rhEPO. In contrast to this standard therapy, the EPO peak concentrations induced by BAY 85-3934 were within the normal physiological range of a factor of approximately 2-fold. Thus, the abnormally high peak concentrations typically associated with standard rhEPO therapy, which may impact on the long-term safety of treatment [3], [50], might be avoided by therapy with BAY 85-3934. The fact that the reticulocyte count was 2-fold higher at days 8 and 15 in animals treated with rhEPO than in those treated with BAY 85-3934 may be due to the timing of measurement, because reticulocyte counts usually reach peak levels 4 days after administration of rhEPO.

BAY 85-3934 was effective in ameliorating and preventing renal anemia in rats after treatment with the nephrotoxic antibiotic gentamicin. This shows that in failing kidneys, responsiveness of the EPO gene to HIF-PH inhibition is preserved, a finding that is in line with previous reports [23], [24], [51]. EPO mRNA expression was induced in failing kidneys by a similar degree, and with comparable sensitivity, to that in healthy control animals. However, the absolute mean expression levels were remarkably reduced, possibly as a consequence of loss of kidney parenchyma. The reduction of EPO expression is believed to be responsible for the occurrence of anemia in the gentamicin model, and in this respect the model resembles the renal anemia observed in patients with CKD [34]. In view of the rapid drop in reticulocyte counts, additional toxic effects of gentamicin on erythropoiesis in the bone marrow are also likely. The data from the expression analyses indicate that hepatic EPO production may significantly contribute to the EPO plasma pool under treatment with BAY 85-3934. Distinct hypoxia-sensitive elements on the EPO gene have been identified as relevant for hepatic EPO expression [52]. However, the liver is not able to compensate for lost renal EPO production without pharmacological intervention. Treatment of patients without functioning kidneys with a HIF-PH-inhibitor has shown that the liver can fully compensate for the lack of renal EPO production [51]. Likewise, hepatic siRNA knock-down of all three HIF-PHs in mice with renal impairment was required to induce sufficient compensatory hepatic EPO production, a requirement that can be replicated by administration of a pan-HIF-PH inhibitor [53].

Systemic inflammation is common in patients with ESRD receiving chronic hemodialysis and may diminish responsiveness to EPO, ultimately presenting as EPO resistance [54], [55]. BAY 85-3934 was effective in correcting inflammation-induced anemia in a rat model of polyarthritis. Whether reduction of monocyte chemotactic protein-1 and hepcidin mRNA expression are hematopoiesis-independent effects of HIF-PH inhibition or result from the correction of anemia cannot be determined based on the current data. Hepatic expression of hepcidin, which is an important pathogenic factor responsible for disturbed iron utilization in inflammatory anemia, is known to be reduced after stimulation of the bone marrow with EPO [56], [57].

The rat model of subtotal nephrectomy shows the key features of CKD – renal impairment, anemia, and hypertension. Notably in this model, and in contrast to treatment with rhEPO, treatment with BAY 85-3934 not only effectively corrected anemia, but also reduced hypertension associated with kidney disease in a dose-dependent manner. The underlying mechanism of this is not clear, but may be related to transcriptional and structural changes in the diseased kidney. Anti-inflammatory and anti-fibrotic changes of transcriptional response were observed in animals of the high dose group but are considered secondary to blood-pressure-normalizing effects rather than a direct effect of BAY 85-3934 on inflammation; however, kidney-protective effects of activation of the HIF-signaling cascade have been postulated [58]–[60].

Antihypertensive therapy is often associated with compensatory increases in renin and activation of the renin–angiotensin–aldosterone system [61]. Paradoxically, following treatment with BAY 85-3934, plasma prorenin levels remained low despite normalization of blood pressure. This effect may provide an additional benefit in the treatment of patients with CKD and anemia. This was in contrast to treatment with enalapril, which showed the expected effect of increasing prorenin. This effect was slightly diminished in combination with BAY 85-3934. Various lines of evidence suggest a link between the up-regulation of HIF and a reduction of blood pressure. Several vasomotor-relevant genes are known members of the HIF target gene cluster [11]. Patients with Chuvash polycythemia, who constitutively express high HIF levels, have been found to have significantly lower blood pressure than matched controls [62]. Furthermore, in transgenic mice with stabilized HIFs, juxtaglomerular cells switch from a renin- to an EPO-secreting cell type [63].

In addition to efficacy in the treatment of anemia, the potential effects of HIF stabilization on long-term health must be considered. HIFs regulate a battery of genes that are important for hypoxia adaptation, and inhibition of HIF-PH may have effects on metabolism and angiogenesis. In the EPO-producing cells of the kidney, the transcriptional activation of EPO is mainly under the control of the PHD2-HIF-2α axis, while in the liver PHD1 and PHD3 also contribute to the oxygen-dependent transcriptional repression of EPO [53], [64]. The rare gain-of-function mutations of HIF-2α and the loss-of-function mutations in the PHD2 gene are associated with benign erythrocytosis [15]. This phenotype can be reproduced in the corresponding transgenic animal models [16], [19], [20]. BAY 85-3934 is a pan-HIF-PH inhibitor, hence potential mode-of-action-dependent implications for safety may be best assessed in view of the phenotype associated with global HIF activation, which is known in patients suffering from Chuvash polycythemia [14]. In addition to severe polycythemia and lowered systemic blood pressure, these patients show changes in muscle metabolism, mild organomegaly, and increased basal tone and hypoxic vasoconstriction of pulmonary blood vessels compared with matched controls. Despite lifelong activation of the HIF system, no increase in the incidence of malignancies has been found. Furthermore, the manifestations of this phenotype would not be expected to arise to the same extent from intermittent activation of the HIF system, and the magnitude of the effect may be comparable to intermittent and moderate high altitude exposure [62], [65], [66].

To explore the transcriptional response after exposure to BAY 85-3934, a panel of HIF target genes was examined, identified in the scientific literature as hypoxia-responsive genes [11], [67]–[69]. CAIX is known to be a reliable histochemical marker of hypoxia and, in our studies, was strongly induced *in vitro*, but was not induced *in vivo* [70]. The expression of HIF target genes was analyzed in kidney and liver samples from rats after exposure to BAY 85-3934 in several settings and at a range of doses. The kidney was chosen as the pharmacological target organ in which EPO response is triggered by the action of the compound. The liver was selected because it is the primary organ of metabolism of this drug. With the exception of EPO, the induction of hypoxia-sensitive gene transcription was found to be absent or comparatively minor, with no fundamental difference between the organs from healthy rats and those from anemia-induced rats, even at doses at which EPO levels were increased by two orders of magnitude.

PHD2 and PHD3 are up-regulated under hypoxia, while PHD1 is hypoxia-insensitive. Interestingly, HIF-dependent transcriptional induction of HIF-PHs has been described as an important negative feedback mechanism that, under hypoxia, compensates for decreased oxygen levels, a mechanism possibly underlying the adaptive modulation of EPO response under prolonged hypoxia [71]. However, in the present studies, the expression of genes encoding HIF-PHs remained unchanged and no adaptive responses were observed after repeated dosing.

Whether the transcriptional changes observed in the preclinical studies translate into corresponding changes at the protein level that are of functional importance can only be answered for the EPO gene, which was the most sensitive gene to treatment with BAY 85-3934. This finding is in line with studies in rodents exposed to hypoxia *in vivo* [18], [48].

In conclusion, the pan HIF-PH inhibitor BAY 85-3934 acts as a hypoxia mimetic *in vivo*, with effective reversion of anemia in animal models of renal and inflammatory anemia. BAY 85-3934 was found to be effective in raising hematocrit levels while stimulating endogenous EPO production within its normal physiological range. Furthermore, BAY 85-3934 can be administered as an oral therapy and, unlike rhEPO, showed antihypertensive effects in a model of CKD. Thus, BAY 85-3934 is an attractive new drug candidate for the treatment of EPO-sensitive anemia, in particular anemia associated with CKD.
